# Analysis of Thromboembolic and Thrombocytopenic Events After the AZD1222, BNT162b2, and MRNA-1273 COVID-19 Vaccines in 3 Nordic Countries

**DOI:** 10.1001/jamanetworkopen.2022.17375

**Published:** 2022-06-14

**Authors:** Jacob Dag Berild, Vilde Bergstad Larsen, Emilia Myrup Thiesson, Toni Lehtonen, Mari Grøsland, Jon Helgeland, Jan Wolhlfahrt, Jørgen Vinsløv Hansen, Arto A. Palmu, Anders Hviid

**Affiliations:** 1 Department of Infection Control and Vaccines, Norwegian Institute of Public Health, Oslo, Norway; 2 Division for Health Services, Norwegian Institute of Public Health, Oslo, Norway; 3 Department of Epidemiology Research, Statens Serum Institut, Copenhagen, Denmark; 4 The Information Services Department, Finnish Institute for Health and Welfare, Helsinki, Finland; 5 The Department of Public Health and Welfare, Finnish Institute for Health and Welfare, Tampere, Finland; 6 Pharmacovigilance Research Center, Department of Drug Design and Pharmacology, University of Copenhagen, Copenhagen, Denmark

## Abstract

**Importance:**

Vaccinations are paramount to halt the COVID-19 pandemic, and safety data are essential to determine the risk-benefit ratio of each COVID-19 vaccine.

**Objective:**

To evaluate the association between the AZD1222, BNT162b2, and mRNA-1273 vaccines and subsequent thromboembolic and thrombocytopenic events.

**Design, Setting, and Participants:**

This self-controlled case series used individual-level data from national registries in Norway, Finland, and Denmark. Participants included individuals with hospital contacts because of coronary artery disease, coagulation disorders, or cerebrovascular disease between January 1, 2020, and May 16, 2021.

**Exposures:**

AZD1222, BNT162b2, or mRNA-1273 vaccine.

**Main Outcomes and Measure:**

Relative rate (RR) of hospital contacts for coronary artery disease, coagulation disorders, or cerebrovascular disease in a 28-day period following vaccination compared with the control period prior to vaccination.

**Results:**

We found 265 339 hospital contacts, of whom 112 984 [43%] were for female patients, 246 092 [93%] were for patients born in 1971 or earlier, 116 931 [44%] were for coronary artery disease, 55 445 [21%] were for coagulation disorders, and 92 963 [35%] were for cerebrovascular disease. In the 28-day period following vaccination, there was an increased rate of coronary artery disease following mRNA-1273 vaccination (RR, 1.13 [95% CI, 1.02-1.25]), but not following AZD1222 vaccination (RR, 0.92 [95% CI, 0.82-1.03]) or BNT162b2 vaccination (RR, 0.96 [95% CI, 0.92-0.99]). There was an observed increased rate of coagulation disorders following all 3 vaccines (AZD1222: RR, 2.01 [95% CI, 1.75-2.31]; BNT162b2: RR, 1.12 [95% CI, 1.07-1.19]; and mRNA-1273: RR, 1.26 [95% CI, 1.07-1.47]). There was also an observed increased rate of cerebrovascular disease following all 3 vaccines (AZD1222: RR, 1.32 [95% CI, 1.16-1.52]; BNT162b2: RR, 1.09 [95% CI, 1.05-1.13]; and mRNA-1273: RR, 1.21 [95% CI, 1.09-1.35]). For individual diseases within the main outcomes, 2 notably high rates were observed: 12.04 (95% CI, 5.37-26.99) for cerebral venous thrombosis and 4.29 (95% CI, 2.96-6.20) for thrombocytopenia, corresponding to 1.6 (95% CI, 0.6-2.6) and 4.9 (95% CI, 2.9-6.9) excess events per 100 000 doses, respectively, following AZD1222 vaccination.

**Conclusions and Relevance:**

In this self-controlled case series, there was an increased rate of hospital contacts because of coagulation disorders and cerebrovascular disease, especially for thrombocytopenia and cerebral venous thrombosis, following vaccination with AZD1222. Although increased rates of several thromboembolic and thrombocytopenic outcomes following BNT162b2 and mRNA-1273 vaccination were observed, these increases were less than the rates observed after AZD1222, and sensitivity analyses were not consistent. Confirmatory analysis on the 2 mRNA vaccines by other methods are warranted.

## Key Points

**Question:**

Are COVID-19 vaccines associated with increased risk of thromboembolic and thrombocytopenic events?

**Findings:**

In this self-controlled case series, AZD1222 was associated with increased rates of cerebral venous thrombosis and thrombocytopenia in 3 Nordic countries. No consistent associations were observed between the mRNA COVID-19 vaccines and coronary artery disease, coagulation disorders and cerebrovascular disease.

**Meaning:**

The findings of this study suggest that AZD1222 vaccination is associated with cerebral venous thrombosis and thrombocytopenia.

## Teaser

This study evaluates the association between the AZD1222, BNT162b2, and mRNA-1273 vaccines and subsequent thromboembolic and thrombocytopenic events between 3 Nordic countries.

## Introduction

Vaccines are considered an important intervention to halt the COVID-19 pandemic. Three different COVID-19 vaccines were introduced into the national vaccination programs in Norway, Finland, and Denmark after their licensure; BNT162b2 mRNA vaccine (Pfizer-BioNTech) in late December 2020, the mRNA-1273 vaccine (Moderna) in January 2021, and the ChAdOx1 nCoV-19 (AZD1222) vaccine (AstraZeneca) in February 2021.

The phase 3 clinical trials did not reveal any major severe adverse events.^1,2,3^ However, in March 2021, several European countries, including Norway, Finland, and Denmark, suspended the use of the AZD1222 because of concerns originating from case reports of unusual and complex thromboembolic pathology with thrombocytopenia.^4,5^ The objective of this study was to evaluate the association between the AZD1222 vaccine and thromboembolic and thrombocytopenic events in Norway, Finland, and Denmark. We also included the BNT162b2 mRNA vaccine and the mRNA-1273 vaccine for comparative purposes.

## Methods

This self-controlled case series analysis followed the Strengthening the Reporting of Observational Studies in Epidemiology (STROBE) reporting guideline. No individual-level data were shared between countries. The study was conducted according to national ethical standards and regulations in each country (eAppendix 3 in the Supplement)

### Data Sources

Using unique personal identification numbers, we linked individual-level data from the national population registries, national patient registers and national vaccination registers separately in Norway, Finland, and Denmark.^6^ In Norway this was done in the emergency preparedness register for COVID-19 (Beredt C19).^7^ Data was extracted during June 2021.

### Exposure

The Norwegian Immunization Register SYSVAK, the Finnish National Vaccination Register, and the Danish Vaccination Register provided data on type of COVID-19 vaccine administered and date of vaccination.^8,9,10^ COVID-19 vaccines are provided free of charge in all countries and are given according to national prioritization guidelines (eAppendix 1 in the Supplement). AZD1222 was primarily used for health care personnel younger than age 65 years in Norway and Denmark. In Finland, AZD1222 was primarily used for adult risk groups aged 18 to 69 years before the suspension. Both mRNA-vaccines have been given to both health care personnel and risk groups, especially the elderly, in all 3 countries. BNT162b2 mRNA vaccine has been most widely used in all 3 countries, while AZD1222 and mRNA-1273 vaccines have been considerably less frequently used. The Ad26.COV2.S vaccine (Johnson & Johnson) has not been used in the national vaccination programs vaccine in Norway, Finland, or Denmark and was not included in this analysis.

### Outcomes

We used the national patient registers to identify hospital visits and admissions because of thromboembolic and thrombocytopenic disease. All 3 countries have national health care coverage for all permanent residents including access to public hospital care with low or no charges.^11,12,13^ The main outcomes were relative rates (RR) of coronary artery disease, coagulation disorders, and cerebrovascular disease (*International Statistical Classification of Diseases and Related Health Problems, Tenth Revision *codes in eAppendix 2 in the Supplement). Secondary outcomes were individual diseases within the main outcome groups of coagulation disorders (venous thrombosis, excluding phlebitis and thrombophlebitis in superficial vessels; arterial thrombosis; disseminated intravascular coagulation; purpura and other hemorrhagic conditions; thrombocytopenia; thrombotic microangiopathy) and cerebrovascular disease (intracranial hemorrhage, cerebral thromboembolic events, and cerebral vein thrombosis). We included all events regardless of type of contact or consultation (ie, in- and outpatient care). Both primary and secondary diagnoses were used.

### Study Population and Follow-up

Our study population included individuals with a study outcome registered as residents in the national population registers per January 1, 2017, and born in 2003 or earlier. We used a washout period of 3 years (2017-2019). Thus, patients with the outcome diagnoses during the washout period were excluded to avoid recording a patient recently diagnosed as an incident case. We also excluded individuals who died or emigrated in the washout period. The study period was January 1, 2020, to May 16, 2021, and only incident cases in this period were included in the analysis. After January 1, 2020, the observation period ended if an individual died or emigrated. To avoid potential confounding from coronavirus disease or other COVID-19 vaccines, the observation period also ended if an individual tested positive for SARS-CoV-2 or was vaccinated with another COVID-19 vaccine.

### Statistical Analysis

Using a common analysis protocol, we conducted separate Norwegian, Finnish, and Danish SCCS analyses to estimate the relative incidences of study outcomes for individuals vaccinated during the study in the 28-day following vaccination (ie, risk period) compared with the prevaccination period (ie, control period) for each vaccine, as well as combined analyses using pooled data from all three countries. The prevaccination period includes 2020 to be able to adjust the model for seasonal variability, by including 4 seasons (March-May, June-August, September-November, December-February). Individuals not vaccinated during the study contribute to this seasonal adjustment. Study participants re-entered the 28-day risk period if they received a second dose. To control for the healthy vaccinee effect, we included a period 14-day prior to vaccination (prerisk period) in the analysis but do not report it as this was not a period of interest. For the same reason, the period after the risk period (ie, 29 days after vaccination and forward) is included in the analysis but not reported. In the SCCS method, each individual acts as their own control, and as such, the model implicitly controls for time-invariant confounders, such as sex, genetics, and chronic underlying health conditions.^14^ We combined the national results using inverse variance weighting. In our analysis, we evaluated the association between several outcomes and exposures (N = 108). Testing multiple hypothesis increases the risk of a false positive association (ie, type I error). We took this into account by calculating adjusted *P* values using the false positive discovery rate calculated by the Benjamini-Hochberg (FDR) method.^15,16^ For all tests, an FDR-adjusted *P* value <.05 was considered statistically significant.

For the main outcomes, we conducted a stratified analysis according to birth cohorts (1971 or earlier, 1972-1986, 1987 or later), sex, and calendar time (before and after March 11, 2021, when the use of AZD1222 was suspended in Denmark and Norway) and conducted several sensitivity analyses to evaluate the robustness of our results and the validity of the assumptions necessary for SCCS analyses. First, we shortened the main risk period to 14 days. Second, we increased the prerisk period to 42 days. Third, we restricted events to hospitalizations with a duration of more than 1 day. Fourth, we used the nominal end date instead of the date of death for individuals dying during the study period. Fifth, we only included cases who survived throughout the study period.

As a post hoc analysis on Finnish and Danish data, we performed a similar analysis using femoral fracture as a negative control outcome. Absolute risk differences cannot be obtained using the SCCS design. However, as a post hoc analysis, we estimated the excess number of events per 100 000 doses for each outcome using a method developed by Wilson et al.^17^

## Results

We found 265 339 incident events during the study period, of which 112 984 events (43%) were among females, 246 092 events (93%) among individuals born 1971 or earlier, 116 931 (44%) were for coronary artery disease, 55 445 (21%) were for coagulation disorders, and 92 963 (35%) were for cerebrovascular disease (Table 1). All outcomes were more common in the older birth cohorts. Coronary artery disease was more common among males, while coagulations disorders and cerebrovascular disease were more evenly balanced across the sexes. These patterns were similar across the 3 countries.

**Table 1.** Characteristics of Thromboembolic and Thrombocytopenic Events in Norway, Finland, and Denmark From January 1, 2020, Through May 16, 2021

Outcome	No. (%)^a^			
	Norway	Finland	Denmark	Combined
**Coronary artery disease**				
Any	34 459 (30)	59 867 (51)	22605 (19)	116 931 (100)
Female	11 513 (33)	22 366 (37)	7898 (35)	41 777 (36)
Male	22 946 (67)	37 501 (63)	14 707 (65)	75 154 (64)
Birth cohort				
1971 or earlier	32 951 (96)	58 328 (97)	21 295 (94)	112 574 (96)
1972 to 1986	1397 (4)	1462 (2)	1212 (5)	4071 (3)
1987 or later	111 (0)	77 (0)	98 (0)	286 (0)
**Coagulation disorders**				
Any	18 280 (33)	18 797 (34)	18 368 (33)	55 445 (100)
Female	8583 (47)	9747 (52)	9132 (50)	27 462 (50)
Male	9697 (53)	9050 (48)	9236 (50)	27 983 (50)
Birth cohort				
1971 or earlier	15 209 (83)	15 391 (82)	15 301 (83)	45 901 (83)
1972 to 1986	1883 (10)	2015 (11)	1888 (10)	5786 (10)
1987 or later	1188 (7)	1391 (7)	1179 (6)	3758 (7)
**Cerebrovascular disease**				
Any	23 121 (25)	38 960 (42)	30 882 (33)	92 963 (100)
Female	10 537 (45)	19 186 (49)	14 022 (45)	43 745 (47)
Male	12 584 (54)	19 774 (51)	16 860 (55)	49 218 (53)
Birth cohort				
1971 or earlier	21 785 (94)	36 697 (94)	29 135 (94)	87 617 (94)
1972 to 1986	1030 (5)	1685 (4)	1366 (4)	4081 (4)
1987 or later	306 (1)	578 (2)	381 (1)	1265 (1)

^a^ Row percentages for “Any”; otherwise, they are column percentages.

During the study period, more than 5.3 million people were vaccinated with either 1 or 2 doses in the 3 countries (Table 2), and 4 265 343 (80%) were vaccinated with BNT162b2, 635 039 (12%) with AZD1222, and 450 723 (8%) with mRNA-1273. Overall, most vaccines were given to people born in 1971 or earlier, except for the AZD1222 vaccine in Norway and Denmark, where more than 50% of the vaccinated were born in 1972 or later. The distribution reflects national prioritization guidelines.

**Table 2.** Characteristics of Vaccinated Individuals by Vaccination Status in Norway, Finland, and Denmark From January 1, 2020, Through May 16, 2021

Characteristics	No. (%)^a^		
	AZD122	BNT162b2	mRNA-1273
**Norway**			
Any	135 743 (9)	1 289 981 (82)	148 816 (9)
Female	105 083 (77)	700 213 (54)	83 157 (56)
Male	30 660 (23)	589 768 (46)	65 659 (44)
Birth cohort			
1971 or earlier	55 092 (41)	1 113 255 (86)	109 853 (74)
1972 to 1986	43 363 (32)	109 853 (9)	24 106 (16)
1987 or later	37 288 (27)	66 873 (5)	14 857 (10)
**Finland**			
Any	358 339 (17)	1 616 402 (75)	176 069 (8)
Female	174 995 (49)	700 875 (43)	79 543 (45)
Male	183 344 (51)	915 527 (57)	96 526 (55)
Birth cohort			
1971 or earlier	327 779 (91)	1 372 238 (85)	156 030 (89)
1972 to 1986	20 505 (6)	166 466 (10)	13 381 (8)
1987 or later	10 055 (3)	77 698 (5)	6658 (4)
**Denmark**			
Any	140 957 (9)	1 358 960 (84)	125 838 (8)
Female	112 716 (80)	748 969 (55)	67 219 (53)
Male	28 241 (20)	609 991 (45)	58 619 (47)
Birth cohort			
1971 or earlier	58 130 (41)	1 246 788 (92)	119 783 (95)
1972 to 1986	46 984 (33)	68 588 (5)	3662 (3)
1987 or later	35 843 (25)	43 584 (3)	2393 (2)
**Combined**			
Any	635 039 (12)	4 265 343 (80)	450 723 (8)
Female	392 794 (62)	2 150 057 (50)	229 919 (51)
Male	242 245 (38)	2 115 286 (50)	220 804 (49)
Birth cohort			
1971 or earlier	441 001 (69)	3 732 281 (88)	385 666 (86)
1972 to 1986	110 852 (17)	344 907 (8)	41 149 (9)
1987 or later	83 186 (13)	188 155 (4)	23 908 (5)

^a^ Row percentages for “Any”; otherwise, they are column percentages.

### AZD1222 Vaccine

In the 28-day risk period following AZD1222 vaccination, we observed 305 events of coronary artery disease, 226 events of coagulation disorders and 231 events of cerebrovascular disease (Table 3). We observed a combined RR for hospital contacts of 2.01 (95% CI, 1.75-2.31) for coagulation disorders and 1.32 (95% CI, 1.16-1.52) for cerebrovascular disease in the 28-day risk period following vaccination with AZD1222 compared with the control period prior to vaccination (Table 3). We did not observe an increased rate of coronary artery disease. We also observed a RR estimate above 1 for several of the secondary outcomes, with a RR of 1.83 (95% CI, 1.56-2.15) for venous thrombosis, 4.29 (95% CI, 2.96-6.20) for thrombocytopenia and 12.04 (95% CI, 5.37-26.99) for cerebral venous thrombosis (CVT). We also observed an increased rate of arterial thrombosis, intracranial hemorrhage, and cerebral thromboembolic events.

**Table 3.** Combined Rate Ratio of Selected Thromboembolic and Thrombocytopenic Outcomes in the 28-Day Period Following Vaccination Compared With the Unvaccinated Period in a Self-controlled Case Series Analysis of AZD1222, BNT162b2, and mRNA-1273, in Norway, Finland, and Denmark From January 1, 2020, Through May 16, 2021

Outcome	AZD1222			BNT162b2			mRNA-1273		
	Cases in the 28-d period following vaccination, No.	Rate ratio (95% CI)	FDR adjusted *P* value	Cases in the 28-d period following vaccination, No.	Rate ratio (95% CI)	*P* value FDR adjusted	Cases in the 28-d period following vaccination, No.	Rate ratio (95% CI)	FDR adjusted *P* value
Coronary artery disease	305	0.92 (0.82-1.03)	.18	3359	0.96 (0.92-0.99)	.03	399	1.13 (1.02-1.25)	.04
Coagulation disorders	226	2.01 (1.75-2.31)	<.001	1674	1.12 (1.07-1.19)	<.001	177	1.26 (1.07-1.47)	.008
Venous thrombosis	174	1.83 (1.56-2.15)	<.001	1394	1.13 (1.07-1.20)	<.001	141	1.21 (1.02-1.44)	.04
Arterial thrombosis	16	2.99 (1.74-5.13)	<.001	123	1.24 (1.02-1.50)	.04	19	2.07 (1.27-3.38)	.007
Disseminated intravascular coagulation	<3	NA	NA	11	NA	NA	<3	NA	NA
Purpura and other heamorrhagic conditions	4	NA	NA	38	1.45 (1.04-2.02)	.04	6	1.89 (0.80-4.45)	.17
Thrombocytopenia	40	4.29 (2.96-6.20)	<.001	172	1.04 (0.88-1.23)	.64	13	0.86 (0.48-1.55)	.64
Thrombotic microangiopathy	<3	NA	NA	6	1.04 (0.44-2.45)	.93	<3	NA	NA
Cerebrovascular disease	231	1.32 (1.16-1.52)	<.001	3228	1.09 (1.05-1.13)	<.001	358	1.21 (1.09-1.35)	.001
Intracranial hemorrhage	35	1.89 (1.33-2.68)	.001	465	1.38 (1.25-1.52)	<.001	61	2.19 (1.67-2.89)	<.001
Cerebral thromboembolic events	194	1.21 (1.05-1.40)	.02	2872	1.06 (1.02-1.11)	.005	313	1.14 (1.01-1.28)	.04
Cerebral venous thrombosis	11	12.04 (5.37-26.99)	<.001	13	1.83 (1.04-3.25)	.05	<3	NA	NA

Abbreviation: FDR, false positive discovery rate by Benjamini-Hochberg correction.

In the stratified analyses, the increase in coagulation disorders and cerebrovascular disease was greater in younger birth cohorts and was only observed after March 11, 2021 (Table 4). Sensitivity analyses were similar to the main analysis and confirmed the robustness of the results (Table 4).

**Table 4.** Stratified and Sensitivity Analyses of Combined Rate Ratio of Selected Thromboembolic and Thrombocytopenic Outcomes in a Self-controlled Case Series Analysis of AZD1222, BNT162b2, and mRNA-1273, in Norway, Finland, and Denmark From January 1, 2020, Through May 16, 2021

Analysis type	Rate ratio (95% CI)								
	Coronary artery disease			Coagulation disorders			Cerebrovascular disease		
	AZD1222	BNT162b2	mRNA-1273	AZD1222	BNT162b2	mRNA-1273	AZD1222	BNT162b2	mRNA-1273
Main analysis^a^	0.92 (0.82-1.03)	0.96 (0.92-0.99)	1.13 (1.02-1.25)	2.01 (1.75-2.31)	1.12 (1.07-1.19)	1.26 (1.07-1.47)	1.32 (1.16-1.52)	1.09 (1.05-1.13)	1.21 (1.09-1.35)
Sex									
Female	1.02 (0.84-1.24)	0.99 (0.94-1.05)	1.16 (0.99-1.37)	2.46 (2.06-2.94)	1.14 (1.06-1.23)	1.23 (0.98-1.53)	1.39 (1.14-1.70)	1.14 (1.08-1.20)	1.27 (1.09-1.49)
Male	0.88 (0.76-1.01)	0.94 (0.89-0.98)	1.12 (0.97-1.28)	1.47 (1.17-1.86)	1.11 (1.03-1.20)	1.29 (1.03-1.60)	1.29 (1.07-1.55)	1.05 (0.99-1.10)	1.18 (1.01-1.38)
Birth cohort									
1971 or earlier	0.93 (0.83-1.05)	0.96 (0.92-1.00)	1.13 (1.02-1.26)	1.80 (1.54-2.11)	1.14 (1.08-1.21)	1.30 (1.11-1.52)	1.24 (1.07-1.43)	1.10 (1.06-1.14)	1.23 (1.10-1.37)
1972-1986	NA	0.69 (0.48-0.97)	NA	2.85 (1.96-4.13)	0.99 (0.76-1.30)	NA	3.23 (2.01-5.19)	0.87 (0.62-1.22)	NA
1987 or later	NA	NA	NA	3.79 (2.29-6.29)	0.71 (0.44-1.14)	NA	NA	NA	NA
Calendar time									
Prior to March 11, 2021	0.92 (0.75-1.12)	0.99 (0.92-1.05)	1.21 (0.99-1.49)	1.18 (0.91-1.54)	1.12 (1.02-1.22)	1.84 (1.35-2.50)	1.20 (0.94-1.55)	1.04 (0.98-1.11)	1.39 (1.13-1.72)
After March 11, 2021	0.93 (0.80-1.09)	0.83 (0.79-0.88)	0.87 (0.74-1.03)	2.22 (1.85-2.65)	1.02 (0.93-1.11)	0.82 (0.62-1.08)	1.28 (1.08-1.51)	1.06 (1.00-1.13)	1.02 (0.85-1.21)
14-d main risk period	0.93 (0.79-1.10)	0.92 (0.87-0.96)	1.18 (1.04-1.35)	1.98 (1.64-2.40)	1.05 (0.99-1.13)	1.04 (0.84-1.29)	1.28 (1.06-1.54)	1.03 (0.98-1.08)	1.20 (1.04-1.38)
Hospitalisations >24 h	0.84 (0.68-1.03)	0.93 (0.88-0.98)	1.06 (0.91-1.23)	2.09 (1.64-2.68)	1.22 (1.13-1.31)	1.73 (1.40-2.14)	1.28 (1.04-1.58)	1.07 (1.02-1.13)	1.20 (1.04-1.38)
42-d pre-risk period	0.93 (0.83-1.05)	0.99 (0.95-1.03)	1.13 (1.01-1.27)	2.04 (1.78-2.35)	1.16 (1.10-1.23)	1.29 (1.08-1.53)	1.33 (1.16-1.52)	1.15 (1.11-1.20)	1.27 (1.12-1.43)
Follow-up to nominal study end date	0.92 (0.82-1.03)	0.95 (0.92-0.99)	1.13 (1.02-1.25)	1.99 (1.73-2.29)	1.11 (1.06-1.17)	1.25 (1.07-1.46)	1.32 (1.16-1.51)	1.08 (1.04-1.13)	1.21 (1.08-1.35)
Exclude cases dying during study	0.88 (0.79-1.00)	0.89 (0.86-0.93)	1.05 (0.95-1.17)	1.88 (1.63-2.17)	1.05 (0.99-1.11)	1.19 (1.02-1.4)	1.16 (1.01-1.34)	0.98 (0.95-1.02)	1.04 (0.93-1.17)

^a^ 28-day risk period compared with unvaccinated follow-up.

### BNT162b2 mRNA Vaccine

In the 28-day risk period following BNT162b2 vaccination, we observed 3359 cases of coronary artery disease, 1674 cases of coagulation disorders, and 3228 cases of cerebrovascular diseases (Table 3). We observed a RR of 1.12 (95% CI, 1.07-1.19) for coagulation disorders and 1.09 (95% CI, 1.05-1.13) for cerebrovascular disease in the 28-day period following vaccination with BNT162b2 (Table 3). We did not observe an increased rate of coronary artery disease. For many secondary outcomes, we observed small rate increases. We observed a small increase in the rate of CVT, but this result was not consistent between the different countries and the increase was smaller compared to that found for AZD1222.

Increased rates of coagulation disorders and cerebrovascular disease were only observed in the oldest birth cohorts, and in the period prior to March 11, 2021, for coagulation disorders and after March 11, 2021, for cerebrovascular disease (Table 4). The results were sensitive to different analytical choices. For example, the RR decreased for all main outcomes and became nonsignificant when we excluded cases dying during the study (Table 4).

### mRNA-1273 Vaccine

In the 28-day risk period following mRNA-1273 vaccination, we observed 399 cases of coronary artery disease, 177 cases of coagulation disorders, and 358 cases of cerebrovascular disease (Table 3). We observed a RR above 1 for all main outcomes; 1.13 (95% CI, 1.02-1.25) for coronary artery disease, 1.26 (95% CI, 1.07-1.47) for coagulation disorders, and 1.21 (95% CI, 1.09-1.35) for cerebrovascular disease (Table 3). For the secondary outcomes, we observed increased rates of most events, but not for thrombocytopenia or purpura and related conditions. We were unable to calculate a RR for CVT because of a low event count.

Almost all the outcomes occurred in people born 1971 or earlier, and we were only able to calculate a RR in this birth cohort (Table 4). The increased rate was only observed in the period prior to March 11, 2021, and the results were sensitive to different analytical choices (Table 4).

### Post Hoc Analysis on Femoral Fracture

We found 23 751 incident cases of femoral fracture in Finland and Denmark (eTable 4 in the Supplement). In the 28-day risk period following vaccination, we observed almost 1300 cases (eTable 5 in the Supplement). We observed a slightly increased rate of femoral fractures following AZD1222-vaccination in Finland, mRNA-1273 vaccination in Denmark, and following BNT162b2 vaccination in both countries.

### Post Hoc Analysis on Absolute Risk

We estimated excess events per 100 000 doses for each outcome (eTable 6 in the Supplement), demonstrating that although the RR is significantly increased, the absolute risk is low. For example, the excess number of thrombocytopenic and CVT events following AZD1222 vaccination are estimated to be 4.9 (95% CI, 2.90-6.90) and 1.6 (95% CI, 0.60-2.60) per 100 000 doses.

## Discussion

In this multinational exploratory self-controlled case series analysis, vaccination with AZD1222 was associated with increased rates of hospital contacts for thromboembolic coagulation disorders, especially for thrombocytopenia. An increased risk of cerebrovascular disease was also observed, in particular for CVT. The increased risk after AZD1222 was consistent across all 3 countries and robust in the sensitivity analyses. We also observed statistically significant increases in the risk of hospital contacts for thrombocytopenic and thromboembolic events after BNT162b2 and mRNA-1273. However, the risk was small compared with those after AZD1222 vaccination. Additionally, the national estimates varied, increased risk were observed only in the oldest cohorts, and sensitivity analysis checking underlying assumptions of the analyses were not consistent. Therefore, the overall and combined increased RR following BNT162b2 and mRNA-1273 vaccination should be interpreted with caution.

Our results for AZD1222 are in line with a comparison of observed and historic rates performed on partly the same population in Norway and Denmark.^18^ In that study, a standardized morbidity ratio of 20.25 (95% CI, 8.14-17.0) was found for CVT and 3.02 (95% CI, 1.76-4.83) for thrombocytopenia after AZD1222 in a study population between 18 and 65 years.

Our results are also aligned with a Scottish national case-control study^19^ on AZD1222 and BNT162b2 that found an association between AZD1222 and idiopathic thrombocytopenic purpura, with an adjusted rate ratio of 5.77 (95% CI, 2.41-13.83).^19^ Furthermore, they also found an increased adjusted rate ratio of arterial thromboembolic and hemorrhagic events after AZD1222 in the case-control study, but they were unable to replicate the results in a post hoc SCCS analysis. The Scottish study did not find an overall increased risk of venous thrombosis, although they observed more events than expected in the younger age groups (16-59 years old). They were unable to analyze CVT specifically because of inadequate power.

The European Medicines Agency has concluded that thrombocytopenia and thrombosis is a rare adverse event following vaccination with AZD1222 and COVID-19 vaccine Janssen,^20^ which is in line with the observed increased RR for thrombocytopenia and CVT following AZD1222 in our study. The absolute risk calculated in our study also indicates the rarity of these events. The mechanism is still under investigation, and it has been suggested that it could be a rare vaccine-related variant of heparin-induced thrombocytopenia because of the presence of antibodies to platelet factor 4.^4,5^ In a study^21^ assessing the coagulation profile of 190 healthy individuals vaccinated with either BNT162b2 or AZD1222 researchers found no hypercoagulability following vaccination. Although it was a small study, it demonstrates that hypercoagulability is not a normal phenomenon after vaccination.

The risk-benefit ratio of the vaccine depends on the risk of contracting COVID-19 and the risk of a severe outcome from COVID-19 weighted against the risk of an adverse event following vaccination. The European Medicines Agency has concluded that the overall risk-benefit ratio remains positive for AZD1222.^22^ Since the risk of a severe outcome from COVID-19 increases with age, several European countries, including Finland, have introduced a lower age-limit for the use of AZD1222 in their national vaccination programs. Denmark and Norway no longer use AZD1222 in their vaccination programs because of adequate availability of alternative vaccines.

A strength of this study is the use of registers with full population coverage in 3 countries with universal health care coverage ensuring equal access to care for all permanent residents. At the end of the study period more than 5.3 million people were vaccinated in the 3 countries. Another strength is the inherent adjustment for time-invariant confounders in the SCCS design, and the resulting control for confounders that can affect the more traditional observational studies when complete data for confounders are not available.

### Limitations

This study has limitations. Our control period includes a period in which declines in health care encounters have been well documented.^23^ For example, if our risk period includes a period of more normal health seeking behavior because of people being vaccinated this could falsely bias our rate ratio upwards. Similarly, the increased awareness following the suspension of AZD1222 in March could have led to surveillance bias, whereby clinically less severe forms of thrombocytopenia might have been more likely to be diagnosed and coded in the registers following vaccination biasing our results upward. In Norway, health care use increased by 66% in the week following March 11, 2021, in those vaccinated with AZD1222.^24^ When we stratified the analysis by date, we observed a significantly increased RR for coagulation disorders and cerebrovascular disease for AZD1222 only after March 11, 2021. This could, to some extent, be due to surveillance bias. However, we observed a similarly high RR when restricting our analysis to cases hospitalized for more than one day (ie, the presumably more severe cases).

A main assumption of the SCCS method is that experiencing a study event does not influence the subsequent probability of getting vaccinated. This bias is difficult to adjust for, and therefore, we included a post hoc negative control outcome. Femoral fracture is especially common in elderly individuals and may result in long-term incapacity which may affect the probability of being vaccinated. We found an association of femoral fracture with COVID-19 vaccinations, up to a RR of 2, cautioning that the SCCS method may yield weak but positive associations because of this bias in our setting. Thus, any positive low-grade association for a serious outcome that may result in long-term incapacity should be interpreted with caution.

Another main assumption is that the occurrence of the event of interest must not affect the observation period. For the more serious events, especially those resulting death, this is likely violated and may result in bias toward positive findings. The RR for both BNT162b2 and mRNA-1273 diminished when we removed cases of individuals who died during the study period, indicating a possible violation of this assumption. The increased RR was observed only in the oldest birth cohort and in the period before March 11, 2021, in which the most fragile population was vaccinated. We speculate that the natural death rate in the oldest birth cohort may bias the main results for the 2 mRNA vaccines upwards.

Although the SCCS analysis automatically adjusts for constant, individual factors, it is possible that risks increase during the study period for the very oldest. Therefore, the marginally increased RR above 1 for the combined results for BNT162b2 and mRNA-1273 should be interpreted with caution. We do not think our analysis on AZD1222 is affected in the same way because it was primarily used for health care personnel younger than 65 years in Norway and Denmark and in people younger than 70 years in Finland.

In our study, we included broad and heterogeneous categories of related diseases to enable capturing any general tendency for blood clotting and thrombocytopenia not captured by specific diagnoses alone. However, these categories may be more difficult to interpret clinically in contrast to specific diagnoses, which we also include in our study.

## Conclusions

In this study, we observed an increased rate of hospital contacts because of coagulation disorders and cerebrovascular disease, especially for thrombocytopenia and CVT, after AZD1222 vaccination. Although increased rates of several thromboembolic and thrombocytopenic outcomes after BNT162b2 and mRNA-1273 vaccination were observed, these increases were smaller than those observed after AZD1222, and sensitivity analyses were not consistent. Confirmatory analysis on the 2 mRNA vaccines by other methods are warranted.

## Notes

Supplement. **eTable 1. **National and combined rate ratio of selected thromboembolic and thrombocytopenic outcomes in the 28-day period following vaccination compared to the unvaccinated period in a self-controlled case series analysis of the AZD1222 vaccine in Norway, Finland, and Denmark from January 1, 2020, through May 16, 2021 **eTable 2. **National and combined rate ratio of selected thromboembolic and thrombocytopenic outcomes in the 28-day period following vaccination compared with the unvaccinated period in a self-controlled case series analysis of the BNT162b2 mRNA vaccine in Norway, Finland, and Denmark from January 1, 2020, through May 16, 2021 **eTable 3. **National and combined rate ratio of selected thromboembolic and thrombocytopenic outcomes in the 28-day period following vaccination compared with the unvaccinated period in a self-controlled case series analysis of the mRNA-1273 vaccine in Norway, Finland, and Denmark from January 1, 2020, through May 16, 2021 **eTable 4. **Characteristics of femoral fracture events from Finland and Denmark from January 1, 2020, through May 16, 2021 **eTable 5.** Rate ratio of femoral fracture events in a self-controlled case series analysis of AZD1222, BNT162b2 and mRNA-1273, in Finland and Denmark from January 1, 2020, through May 16, 2021 **eTable 6.** Excess events of selected thromboembolic and thrombocytopenic outcomes per 100 000 doses among vaccinated individuals in a self-controlled case series analysis of the AZD1222, BNT162b2 and mRNA-1273 vaccines in Norway, Finland, and Denmark from January 1, 2020, through May 16, 2021 **eAppendix 1.** Prioritization of vaccines per country **eAppendix 2.** *International Statistical Classification of Diseases and Related Health Problems, Tenth Revision* used to define study outcomes **eAppendix 3.** Ethics
