# Supplementary material for: Analysis of Thromboembolic and Thrombocytopenic Events After the AZD1222, BNT162b2, and MRNA-1273 COVID-19 Vaccines in 3 Nordic Countries
Source: JAMA Netw Open. 2022 Jun 14;5(6):e2217375. doi: 10.1001/jamanetworkopen.2022.17375 (PMC9198750; doi:10.1001/jamanetworkopen.2022.17375)
Supplement: Supplement. — eTable 1. National and combined rate ratio of selected thromboembolic and thrombocytopenic outcomes in the 28-day period following vaccination compared to the unvaccinated period in a self-controlled case series analysis of the AZD1222 vaccine in Norway, Finland, and Denmark from January 1, 2020, through May 16, 2021 eTable 2. National and combined rate ratio of selected thromboembolic and thrombocytopenic outcomes in the 28-day period following vaccination compared with the unvaccinated period in a self-controlled case series analysis of the BNT162b2 mRNA vaccine in Norway, Finland, and Denmark from January 1, 2020, through May 16, 2021 eTable 3. National and combined rate ratio of selected thromboembolic and thrombocytopenic outcomes in the 28-day period following vaccination compared with the unvaccinated period in a self-controlled case series analysis of the mRNA-1273 vaccine in Norway, Finland, and Denmark from January 1, 2020, through May 16, 2021 eTable 4. Characteristics of femoral fracture events from Finland and Denmark from January 1, 2020, through May 16, 2021 eTable 5. Rate ratio of femoral fracture events in a self-controlled case series analysis of AZD1222, BNT162b2 and mRNA-1273, in Finland and Denmark from January 1, 2020, through May 16, 2021 eTable 6. Excess events of selected thromboembolic and thrombocytopenic outcomes per 100 000 doses among vaccinated individuals in a self-controlled case series analysis of the AZD1222, BNT162b2 and mRNA-1273 vaccines in Norway, Finland, and Denmark from January 1, 2020, through May 16, 2021 eAppendix 1. Prioritization of vaccines per country eAppendix 2. International Statistical Classification of Diseases and Related Health Problems, Tenth Revision used to define study outcomes eAppendix 3. Ethics [file jamanetwopen-e2217375-s001.pdf]

Supplemental Online Content

Dag Berild J, Bergstad Larsen V, Myrup Thiesson E, et al. Analysis of thromboembolic and thrombocytopenic events after the AZD1222, BNT162b2, and MRNA-1273 COVID-19 vaccines in 3 Nordic countries. *JAMA Netw Open*. 2022;5(6):e2217375 doi:10.1001/jamanetworkopen.2022.17375

eTable 1. National and combined rate ratio of selected thromboembolic and thrombocytopenic outcomes in the 28-day period following vaccination compared to the unvaccinated period in a self-controlled case series analysis of the AZD1222 vaccine in Norway, Finland, and Denmark from January 1, 2020, through May 16, 2021

eTable 2. National and combined rate ratio of selected thromboembolic and thrombocytopenic outcomes in the 28-day period following vaccination compared with the unvaccinated period in a self-controlled case series analysis of the BNT162b2 mRNA vaccine in Norway, Finland, and Denmark from January 1, 2020, through May 16, 2021

eTable 3. National and combined rate ratio of selected thromboembolic and thrombocytopenic outcomes in the 28-day period following vaccination compared with the unvaccinated period in a self-controlled case series analysis of the mRNA-1273 vaccine in Norway, Finland, and Denmark from January 1, 2020, through May 16, 2021

eTable 4. Characteristics of femoral fracture events from Finland and Denmark from January 1, 2020, through May 16, 2021

eTable 5. Rate ratio of femoral fracture events in a self-controlled case series analysis of AZD1222, BNT162b2 and mRNA-1273, in Finland and Denmark from January 1, 2020, through May 16, 2021

eTable 6. Excess events of selected thromboembolic and thrombocytopenic outcomes per 100 000 doses among vaccinated individuals in a self-controlled case series analysis of the AZD1222, BNT162b2 and mRNA-1273 vaccines in Norway, Finland, and Denmark from January 1, 2020, through May 16, 2021

eAppendix 1. Prioritisation of vaccines per country

eAppendix 2. *International Statistical Classification of Diseases and Related Health Problems, Tenth Revision* used to define study outcomes

eAppendix 3. Ethics

This supplemental material has been provided by the authors to give readers additional information about their work.

© 2022 Dag Berild J et al. *JAMA Network Open.*

eTable 1: National and combined rate ratio of selected thromboembolic and thrombocytopenic outcomes in the 28-day period following vaccination compared with the unvaccinated period in a self-controlled case series analysis of the AZD1222 vaccine in Norway, Finland, and Denmark from January 1, 2020, through May 16, 2021

	Norway		Finland		Denmark		Combined		
Outcome	Rate Ratio (95% CI)	FDR adjusted p-value	Rate Ratio (95% CI)	FDR adjusted p-value	Rate Ratio (95% CI)	FDR adjusted p-value	N	Rate Ratio (95% CI)	FDR adjusted p-value
Coronary artery disease	0.94 (0.64-1.38)	0.908	0.93 (0.82-1.05)	0.512	0.60 (0.30-1.22)	0.254	305	0.92 (0.82-1.03)	0.178
Coagulation disorders	3.29 (2.32-4.67)	<0.001	1.62 (1.37-1.92)	<0.001	3.03 (2.14-4.29)	<0.001	226	2.01 (1.75-2.31)	<0.001
Venous thrombosis	2.36 (1.49-3.73)	0.002	1.54 (1.27-1.86)	<0.001	3.12 (2.13-4.56)	<0.001	174	1.83 (1.56-2.15)	<0.001
Arterial thrombosis	2.11 (0.26-16.92)	0.683	3.11 (1.71-5.69)	0.002	2.80 (0.61-12.81)	0.268	16	2.99 (1.74-5.13)	<0.001
Disseminated intravascular coagulation	-	-	-	-	-	-	< 3	-	-
Purpura and other heamorrhagic conditions	1.72 (0.40-7.42)	0.683	1.22 (0.29- 5.20)	0.913	-	-	4	-	-
Thrombocytopenia	15.23 (7.72-30.05)	<0.001	2.01 (1.24- 3.25)	0.019	7.78 (2.65-22.86)	0.001	40	4.29 (2.96-6.2)	<0.001
Thrombotic microangiopathy	-	-	-	-	-	-	< 3	-	-
Cerebrovascular disease	1.60 (1.01-2.54)	0.086	1.25 (1.08-1.45)	0.018	1.80 (1.18-2.75)	0.017	231	1.32 (1.16-1.52)	<0.001
Intracranial haemorrhage	4.27 (1.73-10.51)	0.004	1.61 (1.08-2.42)	0.064	1.79 (0.54-5.92)	0.449	35	1.89 (1.33-2.68)	0.001
Cerebral thromboembolic events	1.27 (0.74-2.19)	0.605	1.17 (1.00-1.38)	0.132	1.60 (1.00-2.57)	0.097	194	1.21 (1.05-1.4)	0.017
Cerebral venous thrombosis	31.29 (5.05-193.77)	0.002	7.97 (2.70-23.53)	0.002	14.27 (2.82-72.32)	0.005	11	12.04 (5.37-26.99)	<0.001

FDR: false positive discovery rate by Benjamini-Hochberg correction.

© 2022 Dag Berild J et al. *JAMA Network Open.*

eTable 2: National and combined rate ratio of selected thromboembolic and thrombocytopenic outcomes in the 28-day period following vaccination compared with the unvaccinated period in a self-controlled case series analysis of the BNT162b2 mRNA vaccine in Norway, Finland, and Denmark from January 1, 2020, through May 16, 2021

	Norway		Finland		Denmark		Combined		
Outcome	Rate Ratio (95% CI)	FDR adjusted p-value	Rate Ratio (95% CI)	FDR adjusted p-value	Rate Ratio (95% CI)	FDR adjusted p-value	N	Rate Ratio (95% CI)	FDR adjusted p-value
Coronary artery disease	1.08 (1.02-1.14)	0.021	0.90 (0.84- 0.96)	0.004	0.83 (0.76-0.89)	<0.001	3359	0.96 (0.92-0.99)	0.033
Coagulation disorders	1.16 (1.07-1.27)	0.002	1.03 (0.92- 1.14)	0.812	1.15 (1.06-1.25)	0.005	1674	1.12 (1.07-1.19)	<0.001
Venous thrombosis	1.18 (1.07-1.30)	0.003	1.04 (0.92- 1.17)	0.700	1.15 (1.05-1.26)	0.007	1394	1.13 (1.07-1.2)	<0.001
Arterial thrombosis	1.56 (1.19-2.05)	0.004	0.85 (0.53- 1.34)	0.662	1.03 (0.72- 1.48)	0.992	123	1.24 (1.02-1.5)	0.043
Disseminated intravascular coagulation	3.43 (1.60-7.39)	0.004	-	-	6.08 (1.16-31.79)	0.069	11	-	-
Purpura and other heamorrhagic conditions	1.51 (0.93-2.46)	0.163	0.65 (0.24- 1.80)	0.642	1.70 (1.02- 2.85)	0.085	38	1.45 (1.04-2.02)	0.041
Thrombocytopenia	0.99 (0.80-1.22)	0.972	1.20 (0.83- 1.74)	0.586	1.06 (0.73- 1.54)	0.946	172	1.04 (0.88-1.23)	0.642
Thrombotic microangiopathy	0.88 (0.26-2.97)	0.924	2.02 (0.24-17.07)	0.690	0.96 (0.22-4.23)	0.998	6	1.04 (0.44-2.45)	0.934
Cerebrovascular disease	1.11 (1.04-1.18)	0.007	1.12 (1.05- 1.20)	0.004	1.05 (0.99-1.12)	0.222	3228	1.09 (1.05-1.13)	<0.001
Intracranial haemorrhage	1.34 (1.13-1.58)	0.003	1.30 (1.08- 1.56)	0.019	1.48 (1.26-1.74)	<0.001	465	1.38 (1.25-1.52)	<0.001
Cerebral thromboembolic events	1.09 (1.02-1.17)	0.025	1.10 (1.02- 1.17)	0.039	1.01 (0.94-1.08)	0.990	2872	1.06 (1.02-1.11)	0.005
Cerebral venous thrombosis	2.59 (1.01-6.64)	0.086	0.55 (0.13- 2.28)	0.642	2.11 (0.92- 4.85)	0.139	13	1.83 (1.04-3.25)	0.045

FDR: false positive discovery rate by Benjamini-Hochberg correction.

© 2022 Dag Berild J et al. *JAMA Network Open.*

eTable 3: National and combined rate ratio of selected thromboembolic and thrombocytopenic outcomes in the 28-day period following vaccination compared with the unvaccinated period in a self-controlled case series analysis of the mRNA-1273 vaccine in Norway, Finland, and Denmark from January 1, 2020, through May 16, 2021

	Norway		Finland		Denmark		Combined		
Outcome	Rate Ratio (95% CI)	FDR adjusted p-value	Rate Ratio (95% CI)	FDR adjusted p-value	Rate Ratio (95% CI)	FDR adjusted p-value	N	Rate Ratio (95% CI)	FDR adjusted p-value
Coronary artery disease	1.34 (1.13-1.59)	0.003	1.03 (0.88-1.21)	0.857	0.98 (0.77-1.26)	0.992	399	1.13 (1.02-1.25)	0.036
Coagulation disorders	1.56 (1.22-1.99)	0.002	0.76 (0.55-1.05)	0.221	1.35 (1.04-1.76)	0.058	177	1.26 (1.07-1.47)	0.008
Venous thrombosis	1.61 (1.23-2.11)	0.002	0.67 (0.46-0.97)	0.097	1.23 (0.92-1.64)	0.259	141	1.21 (1.02-1.44)	0.041
Arterial thrombosis	1.81 (0.81-4.02)	0.236	1.41 (0.56-3.54)	0.662	3.32 (1.43-7.69)	0.015	19	2.07 (1.27-3.38)	0.007
Disseminated intravascular coagulation	-	-	-	-	-	-	< 3	-	-
Purpura and other heamorrhagic conditions	1.34 (0.31-5.73)	0.871	2.26 (0.51-10.12)	0.541	2.28 (0.51-10.21)	0.390	6	1.89 (0.8-4.45)	0.168
Thrombocytopenia	0.78 (0.31-1.91)	0.760	0.65 (0.20-2.05)	0.662	1.23 (0.43-3.47)	0.897	13	0.86 (0.48-1.55)	0.642
Thrombotic microangiopathy	-	-	-	-	-	-	< 3	-	-
Cerebrovascular disease	1.07 (0.85-1.35)	0.760	1.14 (0.95-1.35)	0.351	1.39 (1.17-1.66)	0.001	358	1.21 (1.09-1.35)	0.001
Intracranial haemorrhage	2.01 (1.18-3.41)	0.022	1.66 (1.08-2.55)	0.064	3.34 (2.06-5.41)	<0.001	61	2.19 (1.67-2.89)	<0.001
Cerebral thromboembolic events	1.03 (0.80-1.32)	0.924	1.08 (0.90-1.31)	0.642	1.27 (1.05-1.54)	0.030	313	1.14 (1.01-1.28)	0.041
Cerebral venous thrombosis	1.23 (0.16-9.69)	0.924	3.63 (0.43-30.63)	0.501	-	-	< 3	-	-

FDR: false positive discovery rate by Benjamini-Hochberg correction.

© 2022 Dag Berild J et al. *JAMA Network Open.*

eTable 4: Characteristics of femoral fracture events from Finland and Denmark from January 1, 2020, through May 16, 2021

	Finland			Denmark		
Femoral fractures	N	(%)		N		(%)
Any	11 538			12 213		
Females	7253	62.86		7887		64.58
Males	4285	37.14		4326		35.42
Birth cohort, –1971	10 754	92.89		11 820		96.78
1972 – 1986	221	1.91		213		1.74
1987 +	563	4.86		180		1.47

© 2022 Dag Berild J et al. *JAMA Network Open.*

eTable 5. Rate ratio of femoral fracture events in a self-controlled case series analysis of AZD1222, BNT162b2 and mRNA-1273, in Finland and Denmark from January 1, 2020, through May 16, 2021

	AZD1222 vaccine				BNT162b2 mRNA vaccine				mRNA-1273 vaccine			
	Finland		Denmark		Finland		Denmark		Finland		Denmark	
Analysis Type	N	Rate Ratio (95% CI)	N	Rate Ratio (95% CI)	N	Rate Ratio (95% CI)	N	Rate Ratio (95% CI)	N	Rate Ratio (95% CI)	N	Rate Ratio (95% CI)
**Main analysis [a]**	39	1.64 (1.18-2.28)	< 3	0.65 (0.09-4.84)	413	1.14 (1.03-1.26)	708	1.50 (1.38-1.62)	60	1.18 (0.9-1.53)	75	2.06 (1.61-2.64)
**Sex**												
Female	24	1.76 (1.15-2.68)	< 3	0.75 (0.10-5.56)	298	1.18 (1.05-1.34)	462	1.40 (1.27-1.54)	45	1.27 (0.94-1.73)	52	2.11 (1.57-2.84)
Male	15	1.48 (0.87-2.51)	< 3	-	115	1.05 (0.86-1.27)	246	1.73 (1.51-1.98)	15	0.95 (0.56-1.61)	23	1.96 (1.26-3.05)
**Birth cohort**												
– 1971	39	1.71 (1.23-2.37)	< 3	0.83 (0.11-6.20)	410	1.14 (1.03-1.27)	699	1.49 (1.38-1.62)	60	1.19 (0.91-1.55)	75	2.08 (1.63-2.67)
1972 – 1986	-	-	< 3	-	3	1.73 (0.51-5.88)	7	2.61 (1.15-5.91)	-	-	< 3	-
1987 +	-	-	< 3	-	-	-	< 3	1.10 (0.26-4.68)	-	-	< 3	-
**Calendar time**												
Prior to March 11	13	1.73 (0.97-3.07)	< 3	-	323	1.23 (1.09-1.38)	333	1.27 (1.13-1.43)	30	1.13 (0.77-1.64)	13	2.36 (1.34-4.17)
After March 11	26	1.59 (1.06-2.37)	< 3	0.50 (0.06-4.24)	90	0.98 (0.79-1.22)	375	1.31 (1.06-1.62)	30	1.24 (0.86-1.8)	62	1.24 (0.67-2.31)
**14-day main risk period**	18	1.47 (0.92-2.36)	< 3	-	231	1.19 (1.04-1.36)	394	1.27 (1.15-1.41)	29	1.10 (0.76-1.6)	44	1.78 (1.30-2.42)
**Hospitalizations > 24 hours**	19	1.93 (1.2-3.1)	< 3	0.89 (0.12-6.66)	202	1.2 (1.04-1.4)	675	1.49 (1.37-1.62)	33	1.22 (0.85-1.74)	72	2.05 (1.60-2.64)
**42-day pre-risk period**	39	1.68 (1.21-2.34)	< 3	0.63 (0.09-4.68)	404	1.16 (1.05-1.29)	552	1.65 (1.51-1.81)	59	1.16 (0.88-1.51)	53	2.49 (1.87-3.33)
**Follow-up to nominal study end date**	39	1.64 (1.18-2.27)	< 3	0.65 (0.09-4.80)	414	1.12 (1.01-1.24)	708	1.45 (1.34-1.57)	60	1.17 (0.9-1.52)	75	2.04 (1.59-2.61)
**Exclude cases dying during study**	37	1.52 (1.09-2.14)	< 3	0.62 (0.08-4.60)	353	0.98 (0.88-1.1)	575	1.21 (1.11-1.33)	51	0.98 (0.74-1.31)	69	1.82 (1.41-2.35)

N: number of cases in the 28-day risk period following vaccination. [a] 28-day risk period compared to unvaccinated follow-up.

© 2022 Dag Berild J et al. *JAMA Network Open.*

eTable 6. Excess events of selected thromboembolic and thrombocytopenic outcomes per 100 000 doses among vaccinated individuals in a self-controlled case series analysis of the AZD1222, BNT162b2 and mRNA-1273 vaccines in Norway, Finland, and Denmark from January 1, 2020, through May 16, 2021

	Excess events (95% CI) per 100 000 doses		
Outcome	AZD122	BNT162b2	mRNA-1273
**Coronary artery disease**	-4.2 (-8.2 - -0.2)	-3.5 (-5.6 - -1.4)	10.8 (3.8 - 17.8)
**Coagulation disorders**	18.1 (14.0 - 22.2)	4.5 (3.0 - 6.0)	8.6 (3.8 - 13.4)
Venous thrombosis	12.6 (9.1 - 16.1)	4.0 (2.6 - 5.4)	5.7 (1.5 - 9.9)
Arterial thrombosis	1.7 (0.5 - 2.9)	0.6 (0.2 - 1.0)	2.3 (0.5 - 4.1)
Disseminated intravascular coagulation	-	-	-
Purpura and other heamorrhagic conditions	-	0.3 (0.1 - 0.5)	0.7 (-0.3 - 1.7)
Thrombocytopenia	4.9 (2.9 - 6.9)	0.2 (-0.3 - 0.7)	-0.5 (-1.7 - 0.7)
Thrombotic microangiopathy	-	0 (-0.1 - 0.1)	-
**Cerebral incidents**	8.9 (5.2 - 12.6)	6.7 (4.6 - 8.8)	14.6 (7.9 - 21.3)
Intracranial haemorrhage	2.6 (1.0 - 4.2)	3.2 (2.4 - 4.0)	7.8 (4.6 - 11.0)
Cerebral thromboembolic events	5.4 (2.1 - 8.7)	4.1 (2.1 - 6.1)	9.0 (2.8 - 15.2)
Cerebral venous sinus thrombosis	1.6 (0.6 - 2.6)	0.1 (-0.1 - 0.3)	-

The excess events are calculated using method described by Wilson and colleagues.[17] The total number of doses are calculated as the total number of exposure days divided by 28. The total days exposed for AZD122, BNT162b2, and mRNA-1273 for all countries were 17543497, 111130207, and 11919997, respectively. 95% confidence intervals are calculated via the delta method.

© 2022 Dag Berild J et al. *JAMA Network Open.*

eAppendix 1. Prioritisation of vaccines per country

<u>Norwegian prioritisation list</u>

1. Residents in nursing homes

2. Age 85 years and above

3. Age 75-84 years

4. Age 65-74 years
AND people between 18¤ and 64 years with these diseases/conditions at high risk of a severe disease course

5. Age 55-64 years with underlying diseases/conditions

6. Age 45-54 years with underlying diseases/conditions

7. Age 18-44 years with underlying diseases/conditions

8. Age 55-64 years

9. Age 45-54 years

10. Age 18-24 years AND 40-44 years

11. Age 25-39 years

Selected groups of healthcare personnel will be vaccinated in parallel with the various priority categories of risk groups.

<u>Danish prioritisation list</u>

1. Residents in nursing homes, assisted living, etc.

2. People aged ≥ 65 who receive both personal care and practical assistance.

3. People aged ≥ 85 years.

4. Personnel in healthcare, elderly care and selected parts of the social sector who are at particular risk of infection or who has been identified as performing a critical function in society.

5. Selected persons with conditions and diseases that result in a significantly increased risk of severe illness from COVID-19.

6. Selected relatives of individuals at significantly increased risk of a severe illness from COVID-19 or relatives who are indispensable as caregivers.

7. People aged 80-84.

8. People aged 75-79.

9. People aged 65-74.

© 2022 Dag Berild J et al. *JAMA Network Open.*

10 A. People aged 60-64 (born between 1957 and 1961).

10 B. People aged 55-59 (born between 1962 and 1966).

10 C. People aged 50-54 (born between 1967 and 1971).

10 D1. People aged 16-19 and 45-49 years (born 1972-1976 and 2002-2005).

10 D2. People aged 20-24 and 40-44 years (born 1977-1981 and 1997-2001).

10 D3. People aged 25-29 and 35-39 years (born 1982-1986 and 1992-1996).

10 D4. People aged 30-34 (born 1987-1991).

Finnish prioritisation list

1. Health care personnel caring for COVID-19 patients, personnel and residents in round-the-clock care units, other social welfare and health care personnel providing urgent care, and election commissions and election commissioners responsible for at-home voting

    1.1. Personnel in intensive care units

    1.2. Personnel in hospital wards and emergency care for diagnosed or suspected COVID-19 patients

    1.3. Personnel at facilities for diagnosed or suspected COVID-19 patients, coronavirus testing personnel, and laboratory personnel involved in coronavirus diagnostics

    1.4. Personnel and residents in social services for residential care and enhanced institutional, round-the-clock care units

    1.5. Other social welfare and health care personnel providing urgent care

    1.6. Election commissions and election commissioners responsible for at-home voting

2. The elderly, and persons with underlying conditions that predispose to severe COVID-19

    2.1. ≥ 80-year-olds, and carers and elderly people living in the same household*

    2.2. 75–79-year-olds, and carers and elderly people living in the same household*

    2.3. 70–74-year-olds, and carers living in the same household

    2.4. 12–69-year-olds with a highly predisposing condition for severe coronavirus disease

    2.5. 12–69-year-olds with a predisposing condition for severe coronavirus disease

3. Others

    3.1. 60–69-year-olds

    3.2. 50–59-year-olds

© 2022 Dag Berild J et al. *JAMA Network Open.*

3.3. 40–49-year-olds

3.4. 30–39-year-olds

3.5. 16–29-year-olds

© 2022 Dag Berild J et al. *JAMA Network Open.*

eAppendix 2. *International Statistical Classification of Diseases and Related Health Problems, Tenth Revision* used to define study outcomes

Outcome (Group (and subgroup))	ICD10 (name)	ICD10 (code)
Coronary artery disease		
	Acute myocardial infarction	I21
	Angina pectoris	I20
	Other acute ischaemic heart diseases	I24
	Atherosclerotic heart disease	I25.1
Coagulation disorders		
Venous trombosis	Pulmonary embolism	I26
	Phlebitis and thrombophlebitis of femoral vein	I80.1
	Phlebitis and thrombophlebitis of other and unspecified deep vessels of lower extremities	I80.2
	Phlebitis and thrombophlebitis of lower extremities, unspecified	I80.3
	Phlebitis and thrombophlebitis of other sites	I80.8
	Phlebitis and thrombophlebitis of unspecified site	I80.9
	Portal vein thrombosis	I81
	Other venous embolism and thrombosis	I82
Arterial trombosis	Arterial embolism and thrombosis	I74
DIC	Disseminated intravascular coagulation	D65
Purpura and other heamorrhagic conditions	Coagulation defect, unspecified	D68.9
	Allergic purpura	D69.0
	Hemorrhagic condition, unspecified	D69.9
Thrombocytopenia	Immune thrombocytopenic purpura (ITP)	D69.3
	Secondary thrombocytopenia	D69.5
	Thrombocytopenia, unspecified	D69.6
Thrombotic microangiopathy	Thrombotic microangiopathy	M311
Cerebrovascular disease		
Intracranial haemorrhage	Subarachnoid haemorrhage	I60
	Intracerebral haemorrhage	I61
	Other nontraumatic intracranial haemorrhage	I62
Cerebral thromboembolic events	Cerebral infarction	I63
	Stroke, not specified as haemorrhage or infarction	I64
	Occlusion and stenosis of precerebral arteries, not resulting in cerebral infarction	I65
	Occlusion and stenosis of cerebral arteries, not resulting in cerebral infarction	I66
	TIA	G45
CVT	Cerebral infarction due to cerebral venous thrombosis, nonpyogenic	I636
	Nonpyogenic thrombosis of intracranial venous system	I676

© 2022 Dag Berild J et al. *JAMA Network Open.*

eAppendix 3. Ethics

Access to the Norwegian data was provided according to the Health Preparedness Act § 2-4 and permitted by the Norwegian Regional Committee for Research Ethics (REK Sør-Øst A, ref. 122745).

Access to the Finnish data was provided according to the Finnish Communicable Diseases Act. THL has the statutory right and legal obligation to monitor safety of vaccines and access necessary information in the national registers.

The Danish study was approved by the Danish Data Protection Agency (Institutional approval reference, 20-1803). Ethical approv

© 2022 Dag Berild J et al. *JAMA Network Open.*
